# Oxidative stress biomarkers and their relationship with cytokine concentrations in overweight/obese pregnant women and their neonates

**DOI:** 10.1186/s12865-016-0184-6

**Published:** 2017-01-07

**Authors:** María Hernández-Trejo, Araceli Montoya-Estrada, Yessica Torres-Ramos, Aurora Espejel-Núñez, Alberto Guzmán-Grenfell, Rosa Morales-Hernández, Maricruz Tolentino-Dolores, Estibalitz Laresgoiti-Servitje

**Affiliations:** 1 Neurobiology of Development Department, Instituto Nacional de Perinatologia, Mexico City, Mexico; 2 Immunobiochemistry Department, Instituto Nacional de Perinatologia, Mexico City, Mexico; 3 Nutrition Research Laboratory, Instituto Nacional de Perinatologia, Mexico City, Mexico; 4 Basic Medical Sciences, TEC-ABC School of Medicine, Tecnologico de Monterrey Carlos Graef Fernandez 154-114, 05120, Mexico City, Mexico

**Keywords:** Oxidative stress, Pregnancy, Obesity, Offspring, Cytokines, Vitamin supplementation, Diabetes, Free fatty acids, Nitrites, Arginase

## Abstract

**Background:**

Oxidative damage present in obese/overweight mothers may lead to further oxidative stress conditions or inflammation in maternal and cord blood samples. Thirty-four pregnant women/newborn pairs were included in this study to assess the presence of oxidative stress biomarkers and their relationship with serum cytokine concentrations. Oxidative stress biomarkers and antioxidant enzymes were compared between the mother/offspring pairs. The presence of 27 cytokines was measured in maternal and cord blood samples. Analyses were initially performed between all mothers and newborns and later between normal weight and mothers with overweight and obesity, and diabetic/non-diabetic women.

**Results:**

Significant differences were found in biomarker concentrations between mothers and newborns. Additionally, superoxide-dismutase activity was higher in pre-pregnancy overweight mothers compared to those with normal weight. Activity for this enzyme was higher in neonates born from mothers with normal pregestational weight compared with their mothers. Nitrites in overweight/obese mothers were statistically lower than in their offspring. Maternal free fatty acids, nitrites, carbonylated proteins, malondialdehyde and superoxide dismutase predicted maternal serum concentrations of IL-4, IL-13, IP-10 and MIP-1β. Arginase activity in maternal plasma was related to decreased concentrations of IL-4 and IL-1β in cord arterial blood. Increased maternal malondialdehyde plasma was associated with higher levels of IL-6 and IL-7 in the offspring.

**Conclusions:**

Oxidative stress biomarkers differ between mothers and offspring and can predict maternal and newborn cytokine concentrations, indicating a potential role for oxidative stress in foetal metabolic and immunologic programming. Moreover, maternal obesity and diabetes may affect maternal microenvironments, and oxidative stress related to these can have an impact on the placenta and foetal growth.

## Background

The presence of obesity is especially relevant among women of reproductive age because of its possible effects on pregnancy. Women with a higher percentage of adipose tissue and a body mass index (BMI) higher than 25 kg/m^2^ before and/or during pregnancy may present a higher incidence of adverse gestational outcomes, including metabolic and anatomic alterations in their offspring [1]. Maternal obesity impacts placental metabolism and foetal redox balance [2] and increases the probability of developing complications during pregnancy, such as preeclampsia and gestational diabetes [3]. Moreover, excess adiposity in the mother may condition metabolic programming of the foetus, which may reduce insulin sensitivity and may favour the development of metabolic syndrome. Babies born from obese mothers have an increased risk of becoming obese or diabetic later in life [4].

Pregnancy associated with maternal obesity is also related to maternal systemic inflammatory conditions and placental inflammation [5], and some authors have shown that it may also condition an inflammatory environment for the foetus [6, 7]. Recently, Aye et al. reported that maternal BMI may be associated with higher maternal cytokine concentrations and activation of placental inflammatory pathways, although they did not find changes in foetal systemic inflammatory cytokines [8]. Pantham et al. reported similar findings in patients with gestational diabetes [5].

The placenta is able to respond to maternal disturbances, playing an important role in stimuli that participate in foetal programming [9]. Systemic cytokines, cortisol levels, hypoxia, and nitrative and oxidative stress are important factors that may participate in foetal metabolic programming [9–12].

Some pregnancy complications, such as preeclampsia and gestational diabetes, may be related to maternal oxidative stress, similar to complications found in small for gestational age newborns who later may develop metabolic syndrome or neurological disorders [13].

Even though foetal programming may be triggered by multiple “insults”, and although their pathophysiological mechanisms have yet to be fully elucidated, oxidative stress in particular has received increased attention. The objective of this study was to assess the presence of oxidative stress biomarkers, the activity of enzymes related to oxidative stress and their relationship with cytokine concentrations in overweight/obese and normal weight, as well as diabetic and non-diabetic, pregnant women and their offspring. Blood samples from mothers and their newborn infants were evaluated for superoxide dismutase, arginase and glutathione peroxidase activity. Levels of free fatty acids, lipid peroxidation and protein carbonylation in plasma were also measured.

## Methods

This study was conducted at the National Institute of Perinatology, a third-level healthcare institution in Mexico City. The hospital’s ethics and research committees approved the study before the participant’s enrolment. All patients signed an informed consent letter, prior to their enrolment in the study.

### Patients and samples

Upon admission to the Labour and Delivery unit, women who met the inclusion criteria (having a single non-complicated pregnancy, who did not receive medication for 3 weeks prior to delivery) and were willing to participate and sign the informed consent form were enrolled in the study. Thirty-four women were included. Obstetric outcomes and drugs administered during delivery were annotated and patients’ clinical and demographic data were retrieved from medical records. Each participant was followed until delivery of the placenta. Maternal blood was obtained by venipuncture prior to delivery, and the newborn’s blood was obtained after placental delivery by umbilical cord arterial puncture under sterile conditions [14]. Blood was collected in heparinized, mineral-free BD Vacutainer-brand tubes and centrifuged at 3500 rpm, and erythrocytes and plasma were aliquoted. Samples were refrigerated immediately for further analysis. Butylated hydrotoluene (0.2 ml) was added as a conservative to the samples in order to evaluate antioxidants, lipids and protein damage.

### Quantitation of lipid hydroperoxides (LOOH) in red blood cell membranes

To evaluate lipid peroxides, we used the assay conditions described by El-Saadani et al. (13). The test solution was mixed with a colour reagent of a commercially available kit for the enzymatic determination of cholesterol (CHO-iodide; Roche). This assay quantifies lipid peroxides by testing their ability to convert iodide to iodine, which can be measured photometrically at 360 nm. Calibration curves were obtained using peroxides such as t-butylhydroperoxide.

### Malondialdehyde (MDA) quantitation

Aliquots of plasma were used to measure MDA at 586 nm, and the values obtained are expressed as pmol carbocyanine per mg dry weight (14). 1-Methyl-2-phenylindole (Sigma-Aldrich, MO) was used as a standard.

### Assessment of carbonylated proteins (CP)

The quantitation of carbonyl groups is one of the most useful markers to determine protein damage. One hundred μl of plasma was combined with 1 ml of 10 mM 2,4-dinitrophenylhydrazine (DNPH) and 2.5 M HCl. Samples were incubated at room temperature under dark conditions and agitated every 15 min during a period of 60 min before being precipitated with 20% trichloroacetic acid (TCA). Samples were then centrifuged for 10 min at 3500 rpm in order to collect the precipitated protein. The pellet was washed with 1 ml of 10% TCA. Subsequently, the precipitate was washed with 3 ml of a mixture of ethylacetate and ethanol (1:1 v/v) in order to eliminate remaining DNPH. The sample was centrifuged and the final precipitate was dissolved in 1 ml of 6 M guanidine chlorhydrate and 20 mM potassium phosphate, and was then incubated for 10 min at 37 °C. Lastly, the products were analysed with a spectrophotometer at a wavelength of 370 nm. The molar extinction coefficient for 2,4-dinitrophenylhydrazine (ε = 22,000/M^−1^ cm^−1^ = 22,000/106 nmol/mL) was used to calculate the carbonyl concentration, which is expressed in nmol of dinitrophenylhydrazine per mg of protein [15].

### Arginase enzymatic activity

Activity of arginase in erythrocyte lysates was evaluated by measuring the release of urea from L-arginine using Corraliza’s spectrophotometric method [16].

### Nitrite quantitation

The concentration of nitrites was quantified in plasma samples through the indirect method of nitric oxide production. Nitrites are considered the final stable product derived from nitric oxide. The anions were measured according to the method described by Miranda [17].

### Free fatty acid (FFA) quantitation

The concentration of free fatty acids was assessed using the colorimetric method developed by Duncombe [18].

### Quantitation of erythrocyte antioxidant enzymes

The quantitative analyses of superoxide dismutase (SOD) and glutathione peroxidase (GSH-Px) in erythrocytes were performed using an enzyme-linked immunosorbent assay (ELISA) from Immulite according to the manufacturer’s requirements and specifications. The results of each assay are reported according to the concentration of serum proteins in mg/dL using Bradford’s method [19].

### Cytokine quantitation

Cytokine concentrations were measured in maternal and umbilical blood serum using a 27-plex panel cytokine assay from Bio-Plex (BioRad).

### Statistical analyses

Results were analysed with SPSS software (IBM, version 22). Data was tested for normality, linearity and homoscedasticity as assumptions for multivariate statistical analyses. No variables required transformation. Statistical analyses performed included one-way analyses of variance, paired and independent samples Student t-tests, multivariate analyses of variance and covariance and multiple linear regression analyses. A probability of α-error < 0.05 was considered to indicate significant results.

## Results

### Descriptive statistics

Thirty-four pregnant women and their offspring were included in the study. Maternal age ranged from 16 to 43 years with a mean age of 30.74 ± 1.35 years. Mean maternal pregestational weight and BMI were 64.36 ± 1.96 kg and 26.38 ± 0.88 kg/m^2^, respectively. The mean weight gain throughout pregnancy was 11.97 ± 1.01 kg. Neonate gestational age at delivery ranged from 34.6 to 41.0 weeks, with a mean of 38.13 ± 0.20 weeks. Mean newborn weight was 3023 ± 57 g, and the mean height was 48.57 ± 0.31 cm. Three infants were born preterm (8.8%).

Fourteen women (41.2%) had a BMI of ≤25 (normal, according to WHO criteria), four women had a pre-pregnancy BMI >30 (obesity) and 16 had a BMI between 25.1 and 29.9 (overweight). Thus, twenty (58.8%) participants were included in the overweight/obesity group because they had a pre-pregnancy BMI higher than 25 kg/m^2^.

Eleven (32.4%) women had been diagnosed with diabetes prior to gestation or developed gestational diabetes (GD); the remaining (67.6%) did not have glucose metabolism disorders. Eight diabetic women belonged to the overweight/obese group and three were in the normal weight group. Only four (11.8%) of the 11 diabetic women had abnormal glucose levels during pregnancy.

The majority of the participants (73.5%) received multivitamin supplementation during pregnancy. Twelve (35.3%) women received drugs with anti-inflammatory properties (metformin and acetyl salicylic acid) at some point during the gestational period.

### Inferential statistics

**Fig. 1:**
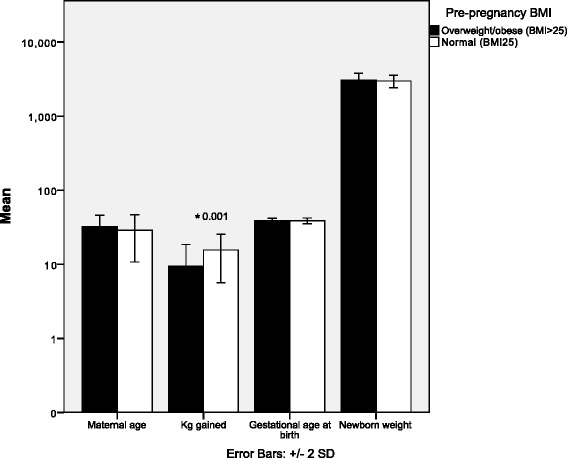
Analysis of variance. Mean differences in maternal age, kg gained during pregnancy, newborn’s gestational age, and newborn weight between overweight/obese mothers and normal-weight mothers

ANOVAs were performed to evaluate differences in maternal age, weight gain during pregnancy, newborn gestational age and newborn weight in normal weight and overweight/obese mothers. Mothers with overweight/obesity gained significantly less weight throughout pregnancy compared to normal weight mothers, which was mainly due to special restriction diets prescribed to overweight mothers. Mean differences and significance of analyses of variance are shown in Fig. 1.

**Fig. 2:**
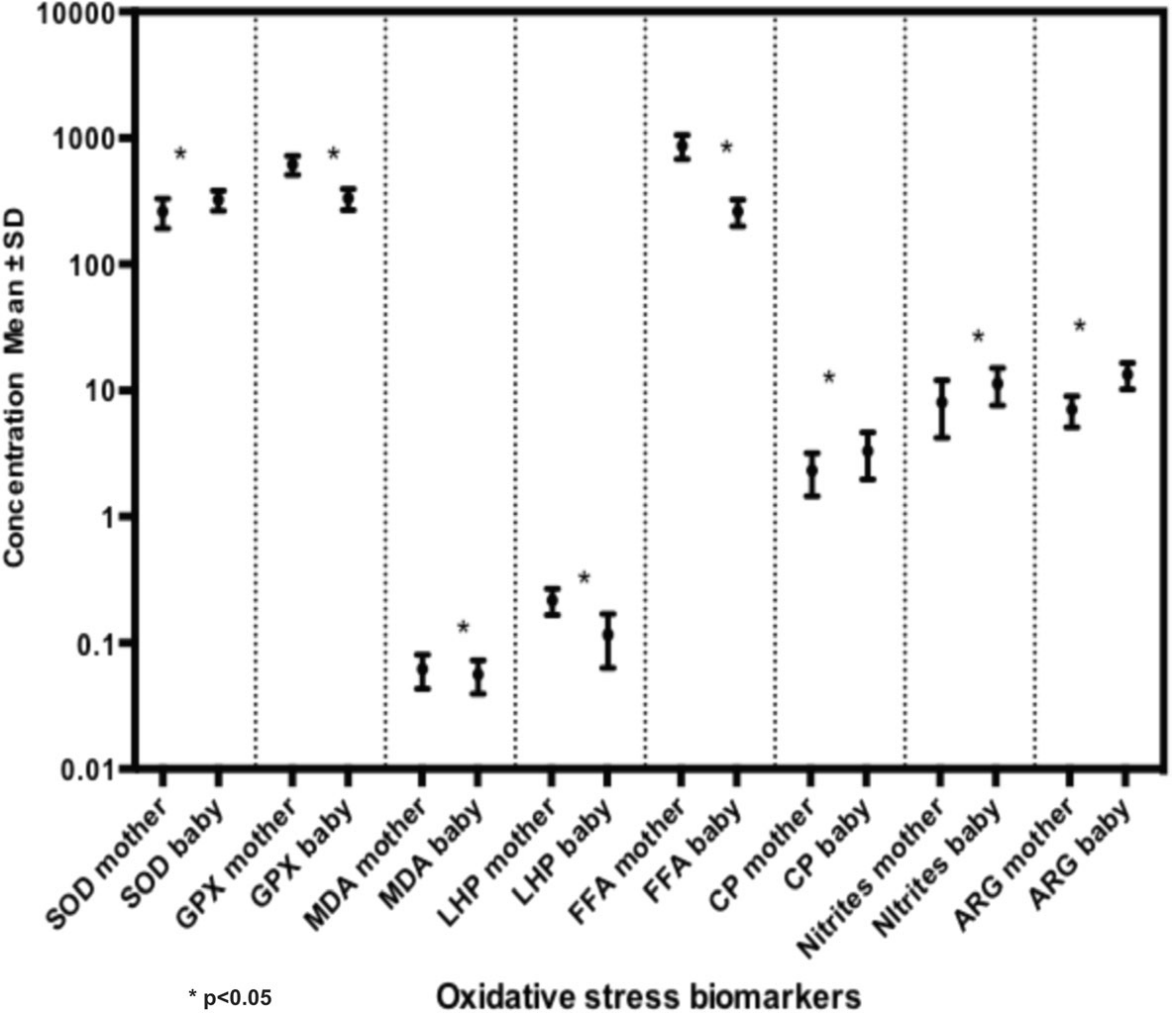
Oxidative stress biomarkers in mother/offspring pairs. Means and standard deviations (*n* = 34). Differences evaluated by paired-samples t Student test. SOD = Superoxide dismutase, GSH-Px = Glutathione peroxidase, MDA = malondialdehyde, LOOH = Lipid-hydroperoxides, FFA = Free fatty acid, CP = Carbonylated proteins. SOD, GSH-Px and ARG values reflect enzymatic activity in erythrocytes (nmol/mg of protein or U/mL)

Paired Student t-tests were used to evaluate the activity of antioxidant enzymes in erythrocytes and the differences in the mean concentrations of oxidative stress biomarkers in all maternal peripheral blood and umbilical arterial blood samples. The 34 mother/offspring pairs were included. Statistical differences were found for all biomarkers that were evaluated (SOD and GSH-Px, MDA, LOOH, FFA, CP, plasma nitrites and arginase activity). SOD and arginase activity, CP and nitrites were significantly higher in umbilical arterial cord blood samples compared with maternal blood samples. Conversely, GSH-Px, MDA, LOOH and FFA were lower in the offspring than in their mothers. Means, mean differences and significance of paired samples t-tests can be found in Table 1 and Fig. 2.

**Table 1:** Oxidative stress biomarkers in maternal venous blood and in arterial umbilical cord blood samples (*n* = 34 pairs)

	Maternal sample Mean (SD)	Umbilical cord sample Mean (SD)	Mean differences	CI 95%	Significance^a^
SOD activity in erythrocytes (U/mL)	261.46 (68.9)	323.82 (59.73)	−62.36	−93.8, −30.9	0.000
GSH-Px activity in erythrocytes (U/mL)	617.36 (106.58)	332.38 (64.09)	284.9	241.4, 328.5	0.000
MDA (nmol carbocianine/mg of dry weight)	0.062 (0.017)	0.055 (0.015)	0.006	0.003, 0.008	0.000
LOOH in red blood membranes (nmol l/mg of dry weight)	0.21 (0.05)	0.11 (0.05)	0.09	0.076, 0.121	0.000
FFA (nmol/mL)	868.63 (188.28)	261.92 (62.49)	606.7	542.3, 671.0	0.000
CP (nmol DNPH/mg of protein)	2.32 (0.87)	3.31 (1.34)	−0.99	−1.43, −0.55	0.000
Nitrites (nmol NO_3_/mL)	8.09 (3.87)	11.29 (3.67)	−3.19	−4.99, −1.39	0.001
Arginase activity in erythrocytes (nmol/mg of protein/min)	7.08 (1.96)	13.29 (3.09)	−6,21	−7.28, −5.14	0.000

^a^Paired-samples t Student test

*SOD* Superoxide dismutase, *GSH-Px* Glutathione peroxidase, *MDA* Malondialdehyde, *LOOH* Lipid-hydroperoxides, *FFA* Free fatty acid, *CP* Carbonylated proteins, *DNPH* dinitrophenylhydrazine

ANOVAs were also performed to evaluate if biomarkers were different in term (gestational age ≥37 weeks, *N* = 31) and preterm newborns (gestational age <37 weeks, *N* = 3). We found that all biomarker concentrations were similar except for malondialdehyde (MDA) that was reduced by 37.5% in term neonates, compared to those born preterm (*p* = 0.025).

To assess if the differences in oxidative stress biomarkers were also present under different maternal conditions, further analyses evaluated differences between groups of mothers, groups of neonates, and between mothers and their offspring. These analyses considered maternal pre-pregnancy BMI, end of gestation BMI, maternal vitamin supplementation, use of drugs with anti-inflammatory activity during pregnancy, the presence of diabetes, and the presence of hyperglycaemia during pregnancy. For the evaluation of mean differences between groups of mothers and groups of neonates, independent samples Student t-tests were performed. To evaluate mean differences between mother and offspring in different conditions, paired samples Student t-tests were used.

As previously mentioned, SOD activity and nitrite concentrations were significantly lower in mothers at the end of pregnancy compared to their newborn infants. However, when comparing these biomarkers and considering pre-pregnancy maternal BMI, significant differences were found in SOD concentrations between women with obesity and women with normal weight (311 U/mL vs 206 U/mL, *p* = 0.019), and between women with overweight and those with normal weight (SOD 289 vs 206 U/mL, *p* = 0.002). Since women with overweight and obesity had similar behaviors in terms of biomarker concentrations, it was decided to group the four women with obesity along with the women in the overweight group. Overall, we found that SOD activity was significantly higher in overweight/obese mothers compared to those with normal weight. This behaviour was not replicated in offspring born to overweight and normal weight mothers as they had similar SOD activity. There was no significant difference in SOD activity in overweight mother/newborn pairs, yet SOD activity was significantly higher in babies from normal weight mothers compared to their mothers. With respect to nitrites, no differences between normal weight and overweight mothers were found. However, in mother/offspring analyses, nitrites in overweight mothers were statistically lower than their babies. Lowest nitrite levels were found in mothers with a BMI ≥ 25.1 kg/m^2^. No differences were found in nitrite concentrations in normal weight mothers and their neonates.

**Fig. 3:**
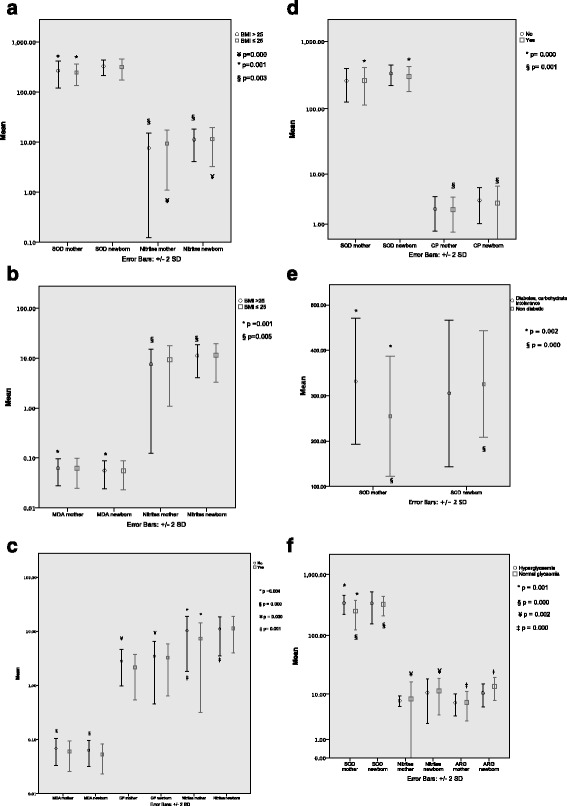
Mean differences of oxidative stress biomarkers in the mother/offspring groups and in mothers vs. neonates, according to BMI, diabetes status, and the use of multivitamins and anti-inflammatory drugs. (*n* = 34 mother/offspring pairs). SOD = Superoxide dismutase (U/mL), CP = Carbonylated proteins (nmol DNPH/mg of protein), Nitrites (nmol/mg protein/min), Arg = Arginase activity (nmol/mg proteins/min), MDA = Malondialdehyde (nmol of carbocyanine/mg of dry weight), NDPH = dinitrophenylhydrazine. **a** Pre-pregnancy BMI. **b** BMI at the end of pregnancy. **c** Multi-vitamin consumption during pregnancy. **d** Use of anti-inflammatories during pregnancy. **e** Diabetes during pregnancy. **f** Hyperglycaemia during pregnancy

Biomarkers were also compared in groups according to BMI at the end of pregnancy. Significant differences in MDA and nitrite concentrations between mothers and their offspring (Table 1) were not found in mothers with normal weight at the end of pregnancy. Moreover, MDA concentration was significantly increased in mothers with a BMI ≥ 25.1 kg/m^2^ at the end of pregnancy compared to their offspring, while nitrite concentration was higher in the offspring in the same group (Fig. 3).

The majority of participants (74%) reported multivitamin consumption during pregnancy. The type, brand and combination of vitamins differed slightly among patients, although all prescribed brands included folic acid. Researchers were not able to collect information regarding the dosage or duration of multivitamin consumption. A dichotomous variable regarding vitamin supplementation (yes/no) was used for analyses. It is unknown why nine participants did not receive vitamin supplementation during pregnancy, as all pregnant women are prescribed multivitamin supplementation according to the hospital’s protocol. Women, who had multivitamin supplementation during pregnancy, as well as their offspring, had non-significantly lower levels of MDA than those participants who did not receive supplementation. Interestingly, MDA concentrations were similar between mothers and babies who did not receive vitamin supplementation, but there were significant differences in MDA concentrations between mothers who were prescribed supplementation and their babies, where MDA was lower in the offspring. Additionally, mothers who received multivitamin supplementation had significantly lower concentrations of CP (reflecting less oxidative stress) compared to those who did not. However, a reduction in CP was not observed in the offspring, as babies from mothers who received vitamin supplementation had significantly higher concentrations of CP than their mothers. This is similar to what was observed in overall concentrations of CP between mothers and newborns in the first analysis shown in Table 1, where babies had significantly more CP than their mothers. Apparently, CP reduction after vitamin supplementation has a relevant impact in the mother but not in the offspring. Regarding nitrites, mothers who received multivitamin supplementation (*n* = 25) had significantly lower nitrite concentrations than those who did not receive supplements. This result was not replicated in the umbilical cord samples.

Drugs with anti-inflammatory properties, such as metformin and acetyl salicylic acid (ASA), were prescribed by physicians to some patients for glycaemic control and preeclampsia prevention, respectively. Paired sample Student t-tests showed that SOD activity was significantly higher in babies from mothers who did not receive metformin or ASA compared to maternal samples. Although without statistical significance, SOD activity was lower in the offspring of women who received metformin or ASA. No differences in SOD activity were observed when comparing maternal samples or cord blood samples between the groups who received these drugs and those that did not. Similarly, CP was higher in cord blood samples compared to maternal samples. CP was non-significantly lower in the neonates of mothers who received metformin or ASA. Babies born from mothers who did not receive drugs with anti-inflammatory activity had significantly higher levels of CP compared with their mothers.

Multivariate analyses of variance and covariance were performed to assess differences in oxidative stress biomarkers in mothers with or without diabetes. Significant differences were found for SOD activity (F(1,31) = 12.053; *p* = 0.002; partial eta squared = 0.28; mean differences 311.06 ± 72.69 vs. 2374.32 ± 52.66 pg/mL) and arginase activity (F(1,31) = 6.39; *p* = 0.017; partial eta squared = 0.17; mean differences 981.37 ± 173.97 vs. 817.07 ± 176.97 pg/mL). Patients with diabetes had increased SOD and arginase activity. However, when controlling for the use of insulin or metformin in these patients, the differences in SOD persisted but a statistically significant difference was not found for arginase in the covariance analysis (F(1,30) = 4.14; *p* = 0.051; partial eta squared = 0.12). Additionally, paired - samples analyses between cord blood samples and maternal samples showed a significantly higher SOD activity in neonates of non-diabetic mothers than their mothers.

From the eleven participants (32.4%) who presented a glucose metabolism disorder during pregnancy, three had pre-gestational diabetes mellitus (PGDM), eight developed gestational diabetes (GD) and 23 were non-diabetic (ND). Differences in all biomarkers were evaluated by performing analyses of variance. Only SOD activity in erythrocytes was found to be significantly higher in mothers with PGDM and GD compared to ND (*p* = 0.045 and 0.035, respectively), and free fatty acid concentration was significantly higher in women with GD compared with ND (Mean Difference = 277.7, CI 95% = 95.6, 459, *p* = 0.004). There were no significant differences in the rest of the biomarkers studied between these three groups.

Even though only four out of the 11 diabetic women who participated in this study had abnormal glucose concentrations throughout pregnancy, the results seem sufficiently strong to show the possible effect of hyperglycaemia on some oxidative stress biomarkers in mothers and their newborns. SOD activity was significantly higher in hyperglycaemic mothers than those mothers with normal glucose levels.

Alternatively, SOD activity was 19% higher in newborns of normoglycaemic mothers compared to their mothers. This difference was not observed when comparing SOD activity between mother and baby in the hyperglycaemic group. Plasma nitrites were significantly higher (28%) in babies born from normoglycaemic mothers compared to those of their mothers. This difference was not found in newborns whose mothers were hyperglycaemic during pregnancy.

In the first minutes of extra-uterine life, newborns had significantly higher arginase activity compared to their mothers. However, this was only shown in offspring of normoglycaemic mothers. Means, mean differences and significance of independent samples and paired samples t-tests between groups can be found in Fig. 3.

Multiple regression analyses were conducted to evaluate which independent variables of maternal oxidative stress (LOOH, MDA, FFA, arginase, CP, nitrites, SOD, GSH-Px) could predict concentrations of 27 different cytokine levels in maternal plasma and in the offspring. Regression analyses indicated that maternal FFA, nitrites, CP, MDA and SOD can affect maternal serum concentrations of IL-4, IL-13, IP-10 and MIP1β. Regression coefficients for significant results are presented in Table 2. Likewise, multiple regression results showed that maternal concentrations of MDA, FFA, LOOH, CP, nitrites, arginase and GSH-Px may predict concentrations of several cytokines in the offspring. Arginase activity in the mother was related to decreased plasma concentrations of IL-4 and IL-1β. Moreover, increased MDA maternal plasma concentrations were associated with higher levels of IL-6 and IL-7 and to lower levels of G-CSF and RANTES in the baby. The presence of maternal serum free fatty acids was also related to lower levels of G-CSF and RANTES in maternal plasma. Regression coefficients for significant results are presented in Table 3.

**Table 2:** Multiple linear regression results for prediction of maternal serum cytokines and chemokines by maternal serum oxidative stress biomarkers (*n* = 34)

Oxidative Stress biomarker^a^ (Maternal plasma)	Cytokine/Chemokine (Maternal plasma)	B	SE	β	Significance	CI 95%
FFA	IL-4	0.003	0.001	0.417	0.028	0.000, 0.006
	MIP-1B	0.031	0.008	0.493	0.001	0.014, 0.048
Nitrites	IP-10	−33.21	15.93	−0.357	0.04	0.799, 1.251
	MIP-1B	0.885	0.402	0.294	0.03	0.056, 1.715
CP	MIP-1B	−6.320	2.003	−0.473	0.004	−10.453, −2.187
MDA	IL-13	−79.02	28.97	−0.534	0.01	−138.82, −19.21
SOD	MIP-1B	−0.068	0.024	−0.399	0.009	−0.117, −0.019

^a^Only significant data is shown. *FFA* Free fatty acids, *CP* Carbonylated proteins, *MDA* Malondialdehyde, *SOD* Superoxide dismutase, *SE* standard error, *β* beta coefficient

**Table 3:** Multiple linear regression results for prediction of umbilical cord blood cytokines and chemokines by maternal serum oxidative stress biomarkers (*n* = 34)

Maternal oxidative stress biomarker	Umbilical cord blood cytokines and chemokines^a^	B	SE_β_	β	Significance	CI 95%
MDA	G-CSF	−909.13	392.25	−0.452	0.02	−1718.7, −99.5
	IP-10	7152.41	2600.86	0.539	0.01	1784.4, 12520.3
	IL-7	−395.88	183.77	−0.491	0.04	−775.1, −16.5
	IL-6	59.55	24.76	0.469	0.02	8.4, 110.6
	IL-5	25.28	10.08	0.497	0.01	4.4, 46.0
	RANTES	−77082.9	28082.5	−0.457	0.01	−13542, −19123
FFA	G-CSF	−0.070	0.031	−0.376	0.03	−0.13, −0.006
	RANTES	−5.612	2.22	−0.358	0.01	−10.2, −1.0
CP	IL-13	0.823	0.348	0.517	0.02	0.1, 1.5
Nitrites	IL-9	417.22	199.89	0.401	0.04	4.6, 829.7
Arginase	IL-4	−0.405	0.154	−0.512	0.01	−0.7, −0.08
	IL-1β	−0.082	0.033	−0.457	0.01	−0.1, −0.01
GSH-Px	RANTES	12.31	4.28	0.447	0.008	3.4, 21.1

^a^Only variables with statistical significance are shown

*SOD* Superoxide Dismutase, *GSH-Px* Glutathion Peroxidase, *MDA* Malondialdehyde, *CP* Carbonylated proteins, *FFA* Free fatty acids, *SE*_*β*_ Coefficient β standard error, *β* Standardized coefficient. *B* Unstandardized regression coefficients

## Discussion

Oxidative stress may be present at the maternal/foetal interphase in early pregnancy. Although it has been implicated in the normal development of the placenta, it may also participate in the pathophysiology of different pregnancy complications [20, 21] because the presence of oxidative stress biomarkers may lead to alterations in the maternal blood supply to the placenta or to inflammation [20].

We found that some oxidative stress biomarkers increased and some decreased in newborns compared with their mothers. SOD, arginase, CP and nitrites were higher in overall cord blood samples compared to maternal samples. In contrast, GSH-Px, MDA, LOOH and FFA levels in neonates were lower than in those of their maternal counterparts. This finding may indicate a buffering role for oxidative stress by the placenta. Giuffrè et al. found that GSH levels were increased in babies born by vaginal delivery, as well as being positively correlated to gestational age [22]. In this study, lower levels of GSH-Px were found in newborns compared with their mothers. Our population may not be comparable to that of Giufreé as gestational age varied in our population, babies were delivered by caesarean section, and many confounding maternal variables were considered within the participants.

Even though overall SOD activity was higher in mothers compared to their babies in this study, SOD was predominantly higher in the group of overweight/obese mothers compared to the normal weight group. Conversely, SOD activity in newborns was higher in those born from normal weight mothers compared to their mothers. Casado et al. also found higher SOD activity in healthy newborns [23]. However, lower SOD activity may also be present in newborns with asphyxia, intrauterine asphyxia and peroxidation conditions [24]. In this study, babies born from overweight/obese mothers had non-significantly lower SOD activity than those born from normal weight mothers. Moreover, increased lipid peroxidation was found in preterm newborns compared to those born at term, a similar finding to previous reports in the literature [25].

Additionally, we found that overweight and obese mothers had the lowest nitrite levels, thus reflecting lower nitric oxide production. Petrella et al. also found lower nitrite production in overweight/obese pregnant women, although these observations were seen in response to L-arginine infusions [26]. In a mouse obesity model, nitrite production was significantly higher in obese vs. lean mice [27]. Other authors report a positive correlation between nitric oxide concentrations and body fat, but a poor correlation with serum lipid concentrations [28], which is different to this study’s findings. However, the previously mentioned results are from non-pregnant women and mice. Given the importance of nitric oxide and nitrites in vascular physiology, their role in overweight/obese pregnant women clearly needs further investigation [9]. Finding low levels of nitric oxide precursors (nitrites) in the plasma of overweight or obese mothers is relevant because none of them developed gestational hypertension or preeclampsia/eclampsia (which was an exclusion criterion in our study). Moreover, this effect was increased when the mother had multi-vitamin consumption during pregnancy. On the other hand, multivitamin supplementation in these patients was also related to a decreased harm to lipids (in the form of MDA) in maternal and cord blood samples. This study’s results support the importance of vitamin supplementation during pregnancy with regard to oxidative stress, as MDA was lower in newborn infants of mothers who had multivitamin supplementation. However, a decrease in CP was only found in the mothers who had supplementation, but not in their babies. Therefore, in our study population, vitamin supplementation protected the mother from protein carbonylation, but not the offspring. The effect of vitamin supplementation in reducing MDA concentrations and protein carbonylation has previously been reported [29].

Oxidative stress has also been linked with the presence of diabetes mellitus [30]. Increased SOD and arginase activity in pregnant diabetic patients were observed in this study. Coughlan et al. also found increased activity of SOD, but also reported increased carbonyl concentrations in women with gestational diabetes [31]. Conversely, Djordjevic et al. found decreased SOD activity in diabetic pregnant women compared to normal pregnant women and non-pregnant women [32]. Arginase has been proposed as a diagnostic biomarker for diabetes mellitus in rat models and in human diabetic subjects because it correlates with blood glucose levels [33, 34]. In this study, we found that differences in arginase activity between diabetic and non-diabetic mothers disappeared when controlling for the effect of the use of metformin and insulin, showing that these may affect arginase activity, similar to a finding by Kashyap et al. [34]. Moreover, Giri et al. found higher arginase activity in the cord blood of gestational diabetic mothers compared to their controls [35]. The offspring of mothers with normal serum glucose, presented higher levels of arginase, compared to their mothers. As this enzyme participates in the regulation of nitric oxide synthesis by reducing the bioavailability of arginine for nitric oxide production [36], this is a relevant finding. Nitric oxide is an important regulator of circulatory changes that occur during fetal life and in the newborn.

Oxidative stress may also modulate cytokine production [37]. Regression analyses in this study showed that maternal FFA was related to higher IL-4 and MIP-1β production in the mother. Additionally, nitrites were associated to increased MIP-1β, but with a decrease in IP-10 concentration. The biomarker in this study that was most associated with inflammatory cytokines was the presence of free fatty acids in pregnant women, which is consistent with the findings of several studies [38]. The relationship between obesity-related biomarkers and inflammatory microenvironments in pregnancy has been described previously [39].

Maternal oxidative stress biomarkers may also be related to changes in cytokine concentrations in newborns. In this study, arginase activity was associated with a down regulation of IL-1β and IL-4.

Although MDA and LOOH were not promoters of inflammatory cytokines in the mother, they were related to the offspring’s inflammatory cytokines. Oxidative stress may influence inflammatory pathways in the newborn, but there is scarce information regarding the role of maternal oxidative stress biomarkers in promoting inflammatory conditions in the offspring. This study shows important differences within oxidative stress biomarkers and the relationship between these and maternal and cord blood cytokines. However, our study presents limitations. Due to the complexity of the sample collection process, only 34 pairs were included in this study. Future studies with larger samples sizes may reveal further information regarding these public health problems.

## Conclusion

Maternal obesity influences oxidative stress and metabolic adjustments during pregnancy, thus affecting the placenta and foetal growth, and may also influence immune system activation. Multivitamin supplementation may protect the mother from oxidative stress, but not necessarily her baby.

Our findings support that obesity and diabetes can affect maternal microenvironments, and that oxidative stress related to these may affect metabolic and immunologic programming of the baby. Further research regarding metabolic and oxidative stress, and the immunological consequences of obesity and gestational diabetes on maternal and newborn health is warranted.

## Abbreviations

ARG: Arginase activity
BMI: Body mass index
CP: Carbonylated proteins
FFA: Free fatty acids
GSH-Px: Glutathione peroxidase
IL: Interleukin
LOOH: Lipid hydroperoxide
MDA: Malondialdehyde
SOD: Superoxide dismutase
